# Is pretreatment assessment of the contralateral breast with MRI useful following a new diagnosis of invasive lobular cancer?

**DOI:** 10.1186/bcr3772

**Published:** 2015-11-05

**Authors:** Harriet Russell, Rebecca Geach, Iain Lyburn, Helen Massey

**Affiliations:** 1 Thirlestaine Breast Centre, Cheltenham, Gloucestershire, UK; 2 Cheltenham Imaging Centre, Cheltenham, Gloucestershire, UK

## Introduction

There is a reported increased incidence of contralateral disease at presentation of invasive lobular cancer (ILC). In our unit breast MRI is undertaken to assess the extent of all newly diagnosed ILC. If mastectomy is planned MRI is still carried out to assess the contralateral breast--we set out to evaluate this.

## Methods

We reviewed 160 reports of consecutive dynamic contrast-enhanced breast MRIs of newly diagnosed ILC (January 2010−June 2015). All cases had been double reported according to the BI-RADS lexicon by two trained readers. We looked at the number of cases of BI-RADS MRM scores of 3 or above in the contralateral breast, second-look ultrasound findings, biopsy rate (U/S or MRI guided) and resultant contralateral cancer detection.

## Results

Of the 160 cases, 23 (14.4%) had an indeterminate or suspicious lesion reported in the contralateral breast. Three of these were contralateral cancers that had already been diagnosed by conventional imaging prior to MRI examination. Seventeen (10.6%) had second-look ultrasound of the contralateral breast: 15 lesions were subsequently biopsied in 11 women. Following negative second-look ultrasounds, two women had MRI-guided biopsy. MRI and subsequent work-up identified three women (1.9%) with previously undiagnosed contralateral malignancies. These were a 5 mm invasive ductal cancer, a 16 mm DCIS and a multicentric ILC.

## Conclusion

The incidence of 'conventional imaging occult' contralateral disease in ILC may be lower than initially reported. The routine use of MRI to assess the contralateral breast is potentially questionable.

